# Supplementary material for: Tissue-resident macrophages are major tumor-associated macrophage resources, contributing to early TNBC development, recurrence, and metastases
Source: Commun Biol. 2023 Feb 3;6:144. doi: 10.1038/s42003-023-04525-7 (PMC9898263; doi:10.1038/s42003-023-04525-7)
Supplement: Supplementary file 2 — Supplementary Information [file 42003_2023_4525_MOESM2_ESM.pdf]

Tissue-resident macrophages are major tumor-associated macrophage resources, contributing to early TNBC development, recurrence, and metastases

Supplementary Information

Ryuichiro Hirano, Koki Okamoto, Miyu Shinke, Marika Sato, Shigeaki Watanabe, Hitomi Watanabe, Gen Kondoh, Tetsuya Kadonosono, Shinae Kizaka-Kondoh

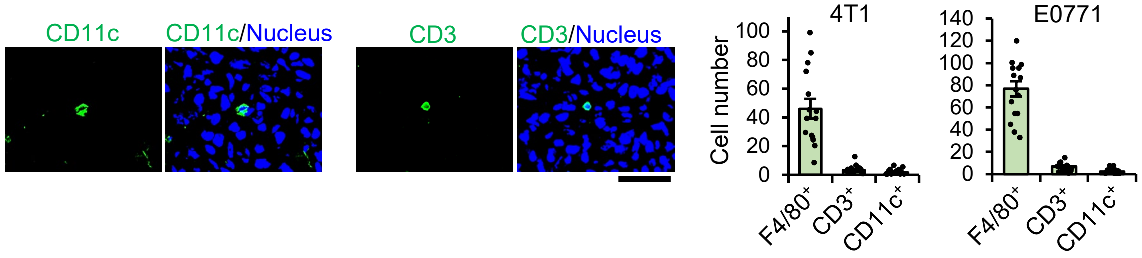

Supplementary Fig 1. Few CD3 and CD11c positive cells in early TNBC.

Sections of 4T1 tumors (~1 mm in diameter) in BALB/c mice 3 days after transplantation. Representative fluorescence images of $CD11c^+$ and $CD3^+$ cells (Left). Scale bar = 50 µm. Average number of F4/80^+, $CD3^+$ cells and $CD11c^+$ cells in a field (Right). n = 15. Error bars indicate standard error of the mean (s.e.m.).

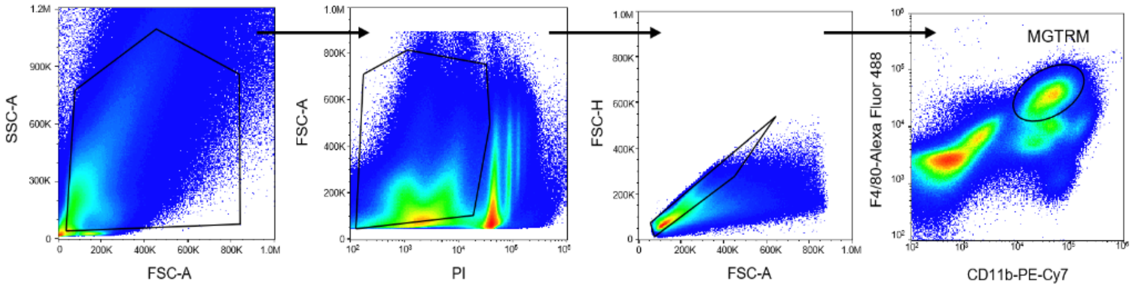

Supplementary Fig 2. Sorting strategy for MGTRMs.

FACS sorting strategy for isolation of MGTRMs from MGFP.

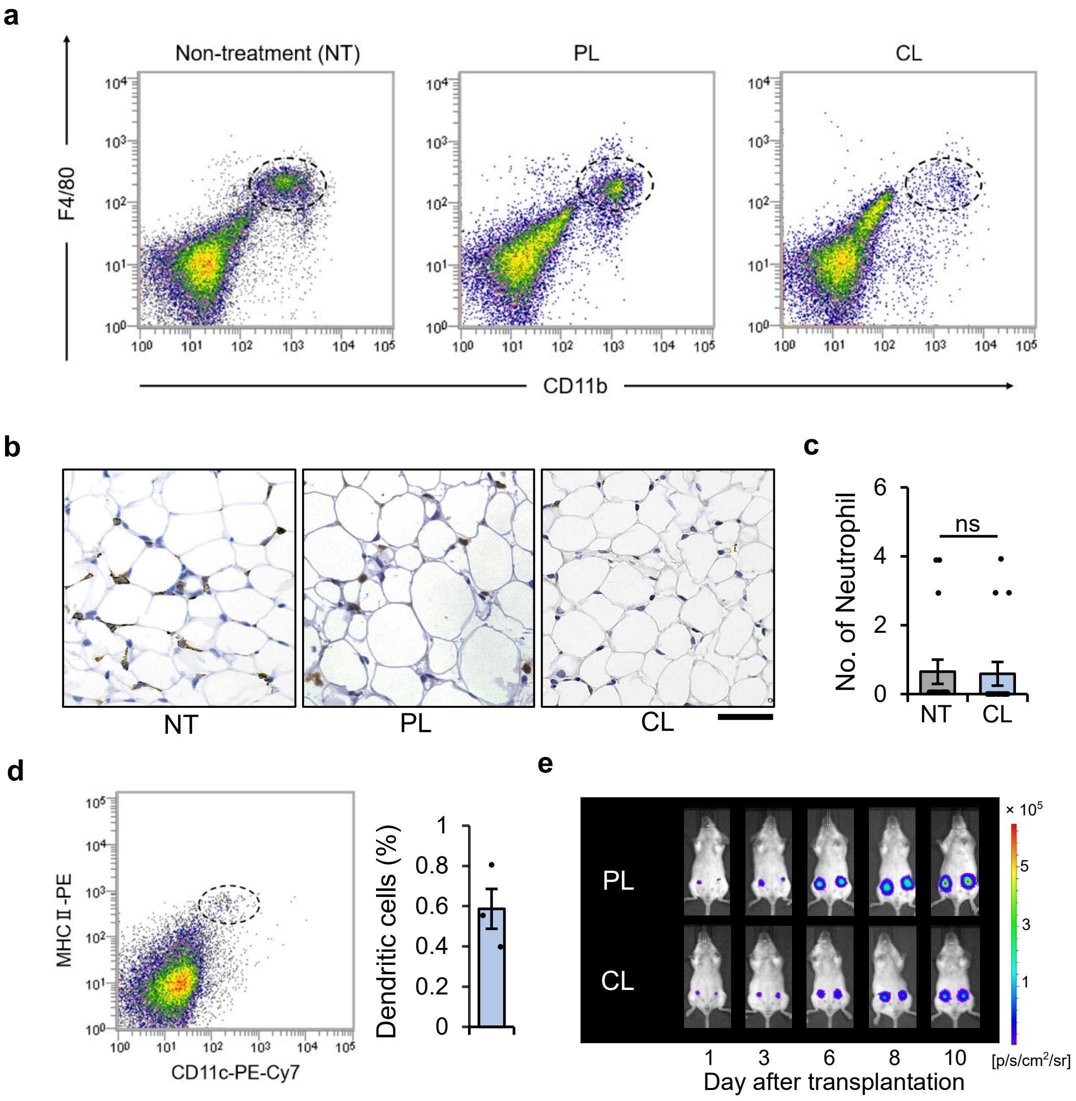

Supplementary Fig 3. The tumor suppressive effect of CL depends on inhibition of MGTRMs.

a Representative flow cytometry plot of CD11b$^+$F4/80$^+$ MGTRMs in tumor-free BALB/c MGFP untreated and 1 days after PL or CL administration. Dot circles indicate MGTRMs.

b Representative immunohistochemical images of F4/80$^+$ cells in tumor-free BALB/c MGFP untreated and 1 days after PL or CL administration. Scale bar is 50 μm.

c The average number in a field of Ly-6G$^+$ neutrophils in tumor-free BLAB/c MGFP untreated (NT) and 1 day after CL administration (CL). n = 17. ns, not significant (*t*-test).

d Representative flow cytometry plot of CD11c$^+$MHC$^+$ dendritic cells (dot cycle) in tumor-free BALB/c MGFP (Left) and % of dendritic cells in MGFP (Right). n = 3.

e Representative bioluminescence images of 4T1 tumors at the indicated days after transplantation in PL- or CL-treated MGFP. **c, d** Error bars indicate s.e.m.

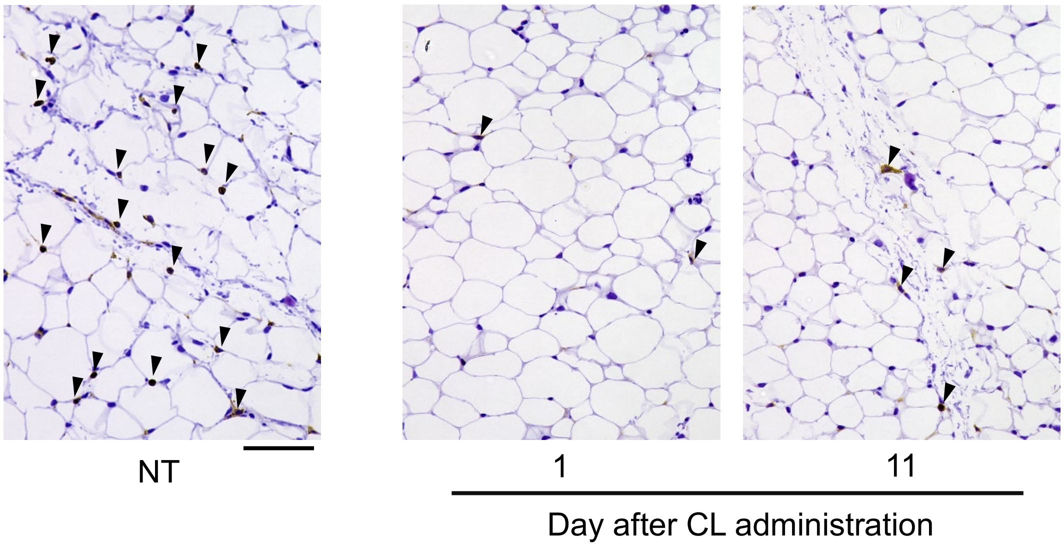

Supplementary Figure 4. Slow recovery of MGTRMs after CL administration

Representative images of F4/80$^+$ cells in BALB/c MGFP untreated, 1 and 11 days after CL administration. Arrow heads indicate F4/80$^+$ cells. Scale bar is 100 μm.

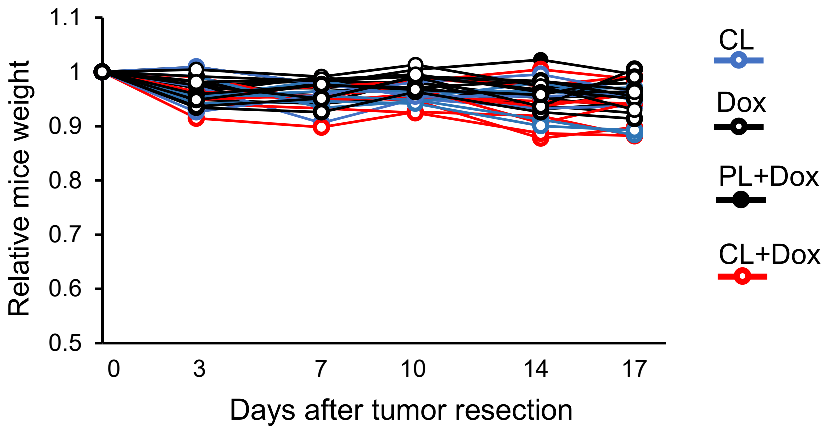

Supplementary Fig 5. The transition of body weight of mice during treatment after surgery.

Body weight of the mice shown in Fig. 5e on the indicated days after tumor resection. There was no significant difference between any groups.
