# Supplementary material for: Tissue-resident macrophages are major tumor-associated macrophage resources, contributing to early TNBC development, recurrence, and metastases
Source: Commun Biol. 2023 Feb 3;6:144. doi: 10.1038/s42003-023-04525-7 (PMC9898263; doi:10.1038/s42003-023-04525-7)
Supplement: Supplementary file 3 — Description of Additional Supplementary Files [file 42003_2023_4525_MOESM3_ESM.pdf]

# Description of Additional Supplementary Files

**File name:** Supplementary Movie 1

**Description:** Photoacoustic imaging movie of 4T1 tumor transplanted PL-treated mammary gland

**File name:** Supplementary Movie 2

**Description:** Photoacoustic imaging movie of 4T1 tumor transplanted CL-treated mammary gland

**File name:** Supplementary Data

**Description:** The source data behind the graphs in the paper
