# Supplementary material for: Tissue-resident macrophages are major tumor-associated macrophage resources, contributing to early TNBC development, recurrence, and metastases
Source: Commun Biol. 2023 Feb 3;6:144. doi: 10.1038/s42003-023-04525-7 (PMC9898263; doi:10.1038/s42003-023-04525-7)
Supplement: Supplementary file 7 — Reporting Summary [file 42003_2023_4525_MOESM7_ESM.pdf]

nature portfolio

Corresponding author(s): Shinae Kizaka-Kondoh

Last updated by author(s): Jan 17, 2023

Reporting Summary

Nature Portfolio wishes to improve the reproducibility of the work that we publish. This form provides structure for consistency and transparency in reporting. For further information on Nature Portfolio policies, see our Editorial Policies and the Editorial Policy Checklist.

Statistics

For all statistical analyses, confirm that the following items are present in the figure legend, table legend, main text, or Methods section.

n/a	Confirmed	
☐	☒	The exact sample size (*n*) for each experimental group/condition, given as a discrete number and unit of measurement
☐	☒	A statement on whether measurements were taken from distinct samples or whether the same sample was measured repeatedly
☐	☒	The statistical test(s) used AND whether they are one- or two-sided<br>*Only common tests should be described solely by name; describe more complex techniques in the Methods section.*
☒	☐	A description of all covariates tested
☒	☐	A description of any assumptions or corrections, such as tests of normality and adjustment for multiple comparisons
☐	☒	A full description of the statistical parameters including central tendency (e.g. means) or other basic estimates (e.g. regression coefficient) AND variation (e.g. standard deviation) or associated estimates of uncertainty (e.g. confidence intervals)
☐	☒	For null hypothesis testing, the test statistic (e.g. *F*, *t*, *r*) with confidence intervals, effect sizes, degrees of freedom and *P* value noted<br>*Give P values as exact values whenever suitable.*
☒	☐	For Bayesian analysis, information on the choice of priors and Markov chain Monte Carlo settings
☒	☐	For hierarchical and complex designs, identification of the appropriate level for tests and full reporting of outcomes
☒	☐	Estimates of effect sizes (e.g. Cohen's *d*, Pearson's *r*), indicating how they were calculated

Our web collection on statistics for biologists contains articles on many of the points above.

Software and code

Policy information about availability of computer code

Data collection	Living image ® 4.7.3 for in vivo imaging by IVIS®-spectrum, ec800 v1.3.6 for flowcytometry, SH800 Software for cell sorting, BZ-X viewer for taking of immunohistochemical image. Photoacoustic imaging system LRK-1 for photoacoustic imaging.
Data analysis	FlowJo 10.8.1 for Flow cytometry analysis. Image J for quantification of fluorescence-positive cells in immunohistochemistry. Pat viewer 3.69 for photoacoustic imaging analysis.

For manuscripts utilizing custom algorithms or software that are central to the research but not yet described in published literature, software must be made available to editors and reviewers. We strongly encourage code deposition in a community repository (e.g. GitHub). See the Nature Portfolio guidelines for submitting code & software for further information.

Data

Policy information about availability of data

All manuscripts must include a data availability statement. This statement should provide the following information, where applicable:

- Accession codes, unique identifiers, or web links for publicly available datasets
- A description of any restrictions on data availability
- For clinical datasets or third party data, please ensure that the statement adheres to our policy

The data generated in this study are available from the corresponding author on reasonable request. The source data underlying Fig. 1a, b, c, e, Fig. 2, Fig. 3, Fig. 4a, b, d, e, f, g, Fig. 5b, c, d, e f, g, Supplementary Fig. S1, Fig. S3c, d, Fig. S5 are provided as Supplementary Data.

Human research participants

Policy information about studies involving human research participants and Sex and Gender in Research.

Reporting on sex and gender	*Use the terms sex (biological attribute) and gender (shaped by social and cultural circumstances) carefully in order to avoid confusing both terms. Indicate if findings apply to only one sex or gender; describe whether sex and gender were considered in study design whether sex and/or gender was determined based on self-reporting or assigned and methods used. Provide in the source data disaggregated sex and gender data where this information has been collected, and consent has been obtained for sharing of individual-level data; provide overall numbers in this Reporting Summary. Please state if this information has not been collected. Report sex- and gender-based analyses where performed, justify reasons for lack of sex- and gender-based analysis.*
Population characteristics	*Describe the covariate-relevant population characteristics of the human research participants (e.g. age, genotypic information, past and current diagnosis and treatment categories). If you filled out the behavioural & social sciences study design questions and have nothing to add here, write "See above."*
Recruitment	*Describe how participants were recruited. Outline any potential self-selection bias or other biases that may be present and how these are likely to impact results.*
Ethics oversight	*Identify the organization(s) that approved the study protocol.*

Note that full information on the approval of the study protocol must also be provided in the manuscript.

Field-specific reporting

Please select the one below that is the best fit for your research. If you are not sure, read the appropriate sections before making your selection.

☒ Life sciences ☐ Behavioural & social sciences ☐ Ecological, evolutionary & environmental sciences

For a reference copy of the document with all sections, see nature.com/documents/nr-reporting-summary-flat.pdf

Life sciences study design

All studies must disclose on these points even when the disclosure is negative.

Sample size	All experiments were performed in triplicate or more and subjected to Student's t-test with reference to papers reporting data using similar experiments. Since mouse experiments tend to have large experimental errors, experiments were performed using 6 or more mice with reference to similar past data.
Data exclusions	No data were excluded from the analyses.
Replication	All have been confirmed to be reproducible by two or more independent experiments. All attempts at replications were successful.
Randomization	In all mouse experiments, mice of the same age were randomly grouped and treated in each experiment. In the local recurrence experiments, mice were grouped so that the amount of residual cancer cells (based on bioluminescence signal intensity) was equal in each treatment group.
Blinding	In reproduction experiments, group allocation and data collection were handled by different people.

Reporting for specific materials, systems and methods

We require information from authors about some types of materials, experimental systems and methods used in many studies. Here, indicate whether each material, system or method listed is relevant to your study. If you are not sure if a list item applies to your research, read the appropriate section before selecting a response.

Materials & experimental systems

n/a	Involved in the study	
☐	☒	Antibodies
☐	☒	Eukaryotic cell lines
☒	☐	Palaeontology and archaeology
☐	☒	Animals and other organisms
☒	☐	Clinical data
☒	☐	Dual use research of concern

Methods

n/a	Involved in the study	
☒	☐	ChIP-seq
☐	☒	Flow cytometry
☒	☐	MRI-based neuroimaging

Antibodies

Antibodies used

For immunohistochemical analysis, α-F4/80 (clone: BM8, supplier: BioLegend, catalog number: 123102), a-SMA (clone: 1A4, supplier: Sigma-Aldrich, catalog number: A2547), α-GFP (clone: polyclonal, supplier: Abcam, catalog number: ab13970), α-CD31 (clone: MEC 13.3, supplier: BD Biosciences, catalog number: 550274), α-FOLR2 (clone: polyclonal, supplier: Novus biologicals, catalog number: NBP2-43654), α-CD206 (clone: polyclonal, supplier: Abcam, catalog number: ab64693), α-CADM1 (clone: 3E1, supplier: MBL, catalog number: CM004-3), α-CD3 (clone: 17A2, supplier: R & D systems, catalog number: MAB4841-100), α-CD11c (clone: N418, supplier: Bio-Rad Laboratories, MCA1369T), anti-rat IgG-Alexa fluor 488 (supplier: Thermo Fisher Scientific, catalog number: A11006), anti-mouse IgG-Alexa fluor 488 (supplier: Thermo Fisher Scientific, catalog number: A11001), anti-chicken IgY-Alexa fluor 647 (supplier: Thermo Fisher Scientific, catalog number: A21449), anti-rabbit IgG-Alexa Fluor 546, (supplier: Thermo Fisher Scientific, catalog number: A10040), anti-hamster IgG- Alexa fluor 488 (supplier: Abcam, catalog number: ab173003).

For flow cytometry, α-CD11b (clone: M1/70, supplier: BioLegend, catalog number: 101216), α-F4/80 (clone CI:A3-1, supplier: Bio-Rad Laboratories, catalog number: MCA497A488T), α-Ly-6c (clone: HK1.4, supplier: BioLegend, catalog number: 128007), α-CD8a (clone: 53-6.7, supplier: eBioscience, catalog number: 12-0081-81), α-NK1.1 (clone: PK136, supplier: eBioscience, catalog number: 12-5941-81), α-CD11c (clone: N418, supplier: eBioscience, catalog number: 25-0114-81), α-MHC2 (clone: M5/114.15.2, supplier: BD Bioscience, catalog number: 557000)

Validation

Antibodies used were purchased with confirmation on the manufacturer's website that they were validated for the species (mouse) and application (immunohistochemistry and flow cytometry).

Eukaryotic cell lines

Policy information about cell lines and Sex and Gender in Research

Cell line source(s)

E0771 and 4T1 murine breast cancer cell lines were obtained from C3H BioSystems and ATCC, respectively. E0771/mKO2-luc2 and 4T1/Fluc cells were established as described previously (Kuchimaru, T. et al., 2018, Nat. Commun.)

Authentication

Create and store a sufficient amount of original stocks separately when purchasing or establishing. At the start of each experiment, cells were recovered from the original stock and used.

Mycoplasma contamination

All cell lines were regularly tested negative for mycoplasma contamination.

Commonly misidentified lines
(See ICLAC register)

No commonly misidentified lines were used.

Animals and other research organisms

Policy information about studies involving animals; ARRIVE guidelines recommended for reporting animal research, and Sex and Gender in Research

Laboratory animals

C57BL/6J (B6), B6(Cg)-Tyrc-2J/J (B6 albino), and BALB/c mice were obtained from Charles River Laboratory Japan. GFP-Tg mice were produced by introducing a fragment of CAAG-GFP into fertilized eggs of B6 mice in Kyoto university. Age-matched females, 8–10 weeks old, were used in all experiments.

Wild animals

No wild animals were used.

Reporting on sex

Because this study was a basic study on a specific subtype of breast cancer, only female mice were used in all mouse experiments.

Field-collected samples

No filed-collected samples were used.

Ethics oversight

All experiments using mice were approved by the Animal Experiment Committees of the Tokyo Institute of Technology and Kyoto University and carried out in accordance with relevant national and international guidelines.

Note that full information on the approval of the study protocol must also be provided in the manuscript.

Flow Cytometry

Plots

Confirm that:

☒ The axis labels state the marker and fluorochrome used (e.g. CD4-FITC).

☒ The axis scales are clearly visible. Include numbers along axes only for bottom left plot of group (a 'group' is an analysis of identical markers).

☒ All plots are contour plots with outliers or pseudocolor plots.

☒ A numerical value for number of cells or percentage (with statistics) is provided.

Methodology

Sample preparation	For preparation of single cell suspension from the mammary gland fat pad, the fourth mammary gland fat pad was removed from 8–10-week-old female mice, minced, and digested in Hank's Balanced Salt Solution containing 500 μg Liberase DH and 0.16 μl DNase I at 37°C for 2 h. Then, a single-cell suspension was obtained by sequentially passing samples through a silk-based membrane with a pore size of 77 μm and strainers with a pore size of 40 μm. After lysing red blood cells with PharmLyse solution for 2 min at room temperature, cells were resuspended in FACS buffer (5% FBS and 2 mM EDTA in PBS). Preparation of single cell suspension from tumors, 4T1 and E0771 tumors were removed from female mice, minced, and digested in 2% FBS/RPMI-1640 containing 500 μg Liberase DH and 0.16 μl DNase I at 37°C for 1 h. A single-cell suspension was then obtained by sequentially passing samples through strainers with pore sizes of 100 and 40 μm. After lysis of red blood cells with PharmLyse solution for 2 min at room temperature, the cells were resuspended in FACS buffer (5% FBS and 2 mM EDTA in PBS).
Instrument	The antibody-labeled cells were sorted and analyzed using an SH800Z fluorescence activated cell sorter and EC800 flow cytometry analyzer, respectively.
Software	FlowJo 10.8.1
Cell population abundance	The percentage of MGTRMs in viable singlet of MGFP in cell sorting was about 17%. We gated viable singlet from the observed all data points and determined the CD11b and F4/80 double positive cells in viable singlet to be MGTRMs.
Gating strategy	From all data points, debris was eliminated with FCS-A and SSC-A, live cells were gated by PI staining with FSC-A, singlet were gated by FSC-A with FSC-H, and target cells were gated with fluorescently labeled antibodies.

☒ Tick this box to confirm that a figure exemplifying the gating strategy is provided in the Supplementary Information.
